# Causalcall: Nanopore Basecalling Using a Temporal Convolutional Network

**DOI:** 10.3389/fgene.2019.01332

**Published:** 2020-01-20

**Authors:** Jingwen Zeng, Hongmin Cai, Hong Peng, Haiyan Wang, Yue Zhang, Tatsuya Akutsu

**Affiliations:** ^1^ School of Computer Science and Engineering, South China University of Technology, Guangzhou, China; ^2^ School of Computer Science, Guangdong Plytechnic Normal University, Guangzhou, China; ^3^ Bioinformatics Center, Institute for Chemical Research, Kyoto University, Kyoto, Japan

**Keywords:** nanopore sequencing, basecalling, deep neural network, temporal convolution, performance comparison, assembly

## Abstract

Nanopore sequencing is promising because of its long read length and high speed. During sequencing, a strand of DNA/RNA passes through a biological nanopore, which causes the current in the pore to fluctuate. During basecalling, context-dependent current measurements are translated into the base sequence of the DNA/RNA strand. Accurate and fast basecalling is vital for downstream analyses such as genome assembly and detecting single-nucleotide polymorphisms and genomic structural variants. However, owing to the various changes in DNA/RNA molecules, noise during sequencing, and limitations of basecalling methods, accurate basecalling remains a challenge. In this paper, we propose Causalcall, which uses an end-to-end temporal convolution-based deep learning model for accurate and fast nanopore basecalling. Developed on a temporal convolutional network (TCN) and a connectionist temporal classification decoder, Causalcall directly identifies base sequences of varying lengths from current measurements in long time series. In contrast to the basecalling models using recurrent neural networks (RNNs), the convolution-based model of Causalcall can speed up basecalling by matrix computation. Experiments on multiple species have demonstrated the great potential of the TCN-based model to improve basecalling accuracy and speed when compared to an RNN-based model. Besides, experiments on genome assembly indicate the utility of Causalcall in reference-based genome assembly.

## Introduction

**Figure 1:**
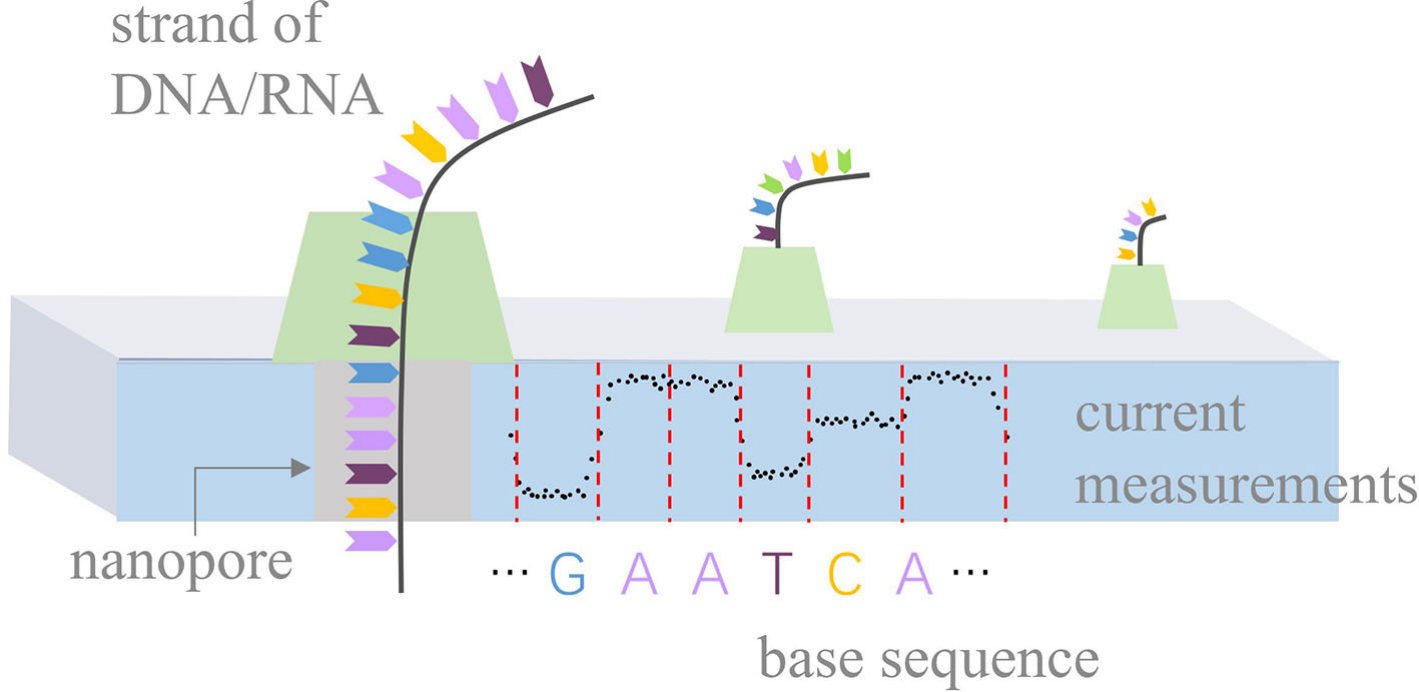
Principle of nanopore sequencing. Biological nanopores are built into an artificial membrane. In the case of DNA, double-stranded DNA is unzipped by a motor protein before passing through the pore. During sequencing, the sequencer measures current changes at a constant frequency with the movement of a DNA strand and stores current measurements in fast5 files. Ideally, current measurements can be divided into events by red dashed lines, according to the movement of *k-mers*.

Nanopore sequencing is a novel third-generation sequencing technology (Leggett and Clark, 2017), focusing on high-throughput, single-molecule, real-time, long-read, and direct DNA/RNA sequencing. It has rapidly developed in recent years and is used in research in a range of biological fields, such as bacterial/viral/plant/human genome assembly and DNA methylation detection (Loman et al., 2015; Quick et al., 2016; Xiao et al., 2017; Michael et al., 2018; Jain et al., 2018; Xiao et al., 2018; Liu et al., 2019). The principle of nanopore sequencing is illustrated in **Figure 1**. The term “nanopore” refers to a nanoscale pore in the sequencer (MinION/GridION/PromethION) with an ionic current passing through it. During sequencing, the sequencer measures changes in current as the DNA/RNA strands pass through the nanopores. A nanopore can hold *k* nucleotides (*k-mer*) simultaneously. For example, k equals 5 for the pore version R9.4. Thus, the current changes indicate the *k-mers* that pass through the nanopores. Such current measurements can be used to identify the base sequences of the DNA/RNA strands.

Basecalling is a fundamental and pivotal step in nanopore sequencing technology. The basecaller is designed to identify the base sequences based on the raw current measurements. The development of basecallers can be roughly divided into two stages. Metrichor and Nanocall (David et al., 2016) are representative basecallers from the first stage, which adopt hidden Markov model (HMM)-based methods. Both of them first convert current measurements to events (with each event corresponding to the movement of a *k-mer* through the pore, as shown in **Figure 1**). Then, they implement an HMM and a Viterbi decoding algorithm to model the event space and further decode the base sequence. Metrichor is the first basecaller provided by Oxford Nanopore Technologies (ONT), and it works in the cloud. Nanocall is a third-party and open-source basecaller. It works offline but only works on data with signal measurements produced under pore version R7.3. In the second stage of basecaller development deep learning-based approaches became popular for basecalling. An example of these is Deepnano (Boža et al., 2017), which uses a bidirectional recurrent neural network (RNN) to model statistical characterizations of events and then predict base sequences. It outperforms Metrichor for the R7.3 platform and demonstrates the potential of RNN in basecalling. Similar implementations are also adopted by official basecallers including Nanonet and Albacore (before v2.0.1). BasecRAWller (Stoiber and Brown, 2017) uses two unidirectional RNNs: one for event boundary prediction and the other for decoding events to a base sequence. In recent research, the efficacy of event-based analyses has been questioned as it heavily relies on the event space. While in practice, it is difficult to precisely convert current measurements to events Chiron (Teng et al., 2018) is an event-free basecaller that directly translates current measurements into a base sequence. It introduces an end-to-end neural network model that combines a convolutional neural network (CNN), an RNN, and a connectionist temporal classification (CTC) decoder (Graves et al., 2006). Wavenano (Wang et al., 2018) adopts the bidirectional WaveNets (Van Den Oord et al., 2016) to predict a *5-mer* label and a move label for each current measurement simultaneously. Based on the segmented *5-mer* label sequences, it decodes the final base sequence using Viterbi decoding algorithm. The official basecallers have also been rapidly updated. Albacore (starting from v2.0.1) also uses an event-free strategy and runs on a CPU. Guppy is similar to Albacore, but benefits from the GPU acceleration. Scrappie is open-source and is used to explore new basecalling approaches. With the release of the latest pore version R10, ONT released Flappie. Flappie uses a flip-flop algorithm to distinguish consecutively repeated bases, which greatly decreases base deletions in homopolymers. Then, Guppy also implements the flip-flop algorithm in the basecalling model. However, the official basecallers (like Albacore and Guppy) are closed-source, which limits our insights into their basecalling methods.

As described above, most recent basecallers, with the exception of Wavenano, use RNN-based models to identify the base sequence based on current measurements. With the recurrent structures, RNN can properly model the time-series data in basecalling. However, in these recurrent structures, the computation of one time point must wait for the result of the former time point. This restricts the speed of RNN-based basecallers like Chiron, when dealing with ultra-long sequencing reads. It also causes difficulty for the RNN-based models in terms of performing parallel computing. A recent study on TCN (Bai et al., 2018) showed that specific convolution architectures perform well in sequence modeling tasks and outperform ordinary RNN in terms of accuracy and execution speed on tasks such as machine translation and audio synthesis. Based on the dilated causal convolution introduced by WaveNets, TCN exhibits extensive receptive fields and thus can properly deal with the long-range temporal dependences required for basecalling. Moreover, the convolution operation is performed on the matrix. This operation not only can accelerate the process of basecalling but also is convenient to perform parallel computing. The advantages outlined above indicate the potential of TCN in accurate and fast basecalling.

In this paper, we present Causalcall, which uses an end-to-end TCN-based model for nanopore basecalling. Causalcall is event-free and designed in the manner of sequence labeling. Therefore, it does not rely on precise label of each current measurement in training. During basecalling, Causalcall directly identifies base segments from the current measurements by using the TCN-based model, and assembles the resulting base segments to generate the final base sequence. Different from the RNN-based model of Chiron, the proposed TCN-based model is much simpler and more efficient. The main contributions of this paper can be summarized as follows:

- We modified the original architecture of TCN and combined it with a CTC decoder. The proposed model could properly model the sequential features of the long-range current measurements and identify the base sequences of varying lengths from the high-dimensional feature space.
- We showed the potential of the TCN-based model in improving the accuracy and speed of basecalling, when compared to the RNN-based model. Experiments on samples lambda phage, *Escherichia coli*, *Klebsiella pneumoniae*, and human showed that Causalcall achieves higher accuracy and is nearly three times faster than RNN-based Chiron.

We hope this work provides a platform for a TCN-based nanopore basecalling playground on which new ideas can flourish.

## Materials and Methods

### Data Preparation

The training set consists of MinION DNA sequencing reads from lambda phage, *E. coli*, and human. The lambda phage and *E. coli* datasets are released by Chiron (Teng et al., 2018). The DNA strands of lambda phage and *E. coli* are sequenced on FLO-MIN106 (pore version R9.4) flow cells using a modified version of the SQK-LSK108 protocol. The reference genome of lambda phage comes from the National Center for Biotechnology Information (NCBI), sequence version NC_001416.1. The reference genome of *E. coli* is assembled by base sequences of *E. coli* samples sequenced by Illumina MiSeq. The human dataset is a subset of human genomic DNA sequencing data released by Nanopore WGS Consortium (Jain et al., 2018). DNA from the GM12878 human cell line is sequenced on FLO-MIN106 flow cells using the SQK-LSK108 protocol. Benefiting from direct sequencing, epigenetic modifications (Zhang et al., 2016) of DNA (e.g., DNA methylation) are preserved. We only selected some of the sequencing data of chromosome 19 for training. GRCh38 with decoys in 1,000 genomes is used as the human reference genome.

To effectively train the network, sequencing data are labeled in the workflow of basecalling, re-squiggle, and segmentation. First, a basecaller is used to identify the base sequence of the sequencing read. Then, the re-squiggle process is performed, involving two steps. In the first step, a base sequence is mapped to the reference genome to obtain the correct sequence. In the second step, the correct sequence is mapped back to the current measurements. By re-squiggling, the alignments between the current boundaries (similar to events) and the actual shifted bases are obtained when a single strand of DNA passes through a pore one base at a time. Finally, the current measurements are divided into segments with a fixed length (*T* = 512). Within the segment, the label sequence is composed of the shifted bases corresponding to the boundaries. Some imprecise alignments between the bases and the current measurements are acceptable due to the sequence-to-sequence learning strategy. In this study, sequencing reads of lambda phage, *E. coli*, and human are basecalled by Albacore v2.3.3 and re-squiggled by Tombo v1.5. To balance the performance of the model, the mixed training set is formed with 13,172 reads from lambda phage, 16,822 reads from *E. coli*, and 13,008 reads from human. The total size of the labeled training data is 86 GB. Additionally, a validation set is used to verify the state of the model during training. This validation set is formed by an additional 10% of reads for each species in the training set.

To comprehensively evaluate the performance of Causalcall, four testing sets are prepared. These testing sets contain sequencing data of lambda phage, *E. coli*, human, and *K. pneumoniae* separately. The lambda phage and *E. coli* datasets contain 5,000 sequencing reads that are randomly selected from the sequencing data described above. These reads are not used for training or validating the model. The human dataset contains 5,000 sequencing reads of human genomic DNA from the Nanopore WGS Consortium, which are randomly selected from the seventh part of the sequencing data of chromosome 13. Sequencing data of *K. pneumoniae* are selected from the benchmarking datasets of a study that aimed to compare the performance of basecallers (Wick et al., 2019). The DNA of *K. pneumoniae* (which contains Dcm-methylation and no plasmids) is sequenced on FLO-MIN106 flow cells using the SQK-LSK108 protocol. It is also sequenced using Illumina HiSeq to generate an accurately assembled genome, which acts as the reference genome of *K. pneumoniae*. We used the dataset of *K. pneumoniae* INF042 as a testing set. It contains the entire sequencing data of the *K. pneumoniae* strain INF042 chromosome (11,278 sequencing reads). As reads of *K. pneumoniae* are not used for training the model, they are used to evaluate the generalization performance of the model. With a sequencing depth of over 100× on average, they are also used to evaluate the assembly accuracy of base sequences from different basecallers.

### Basecalling Workflow

The entire workflow of Causalcall is straightforward, without conversion of current measurements to events. The data pre-processing and post-processing are similar to those of Chiron. However, Causalcall is much simpler and faster by using a more effective end-to-end neural network model. The model is mainly developed on a modified TCN. Thus, it can process all current measurements in a long time series simultaneously. As noise during sequencing typically appears as outliers in the current signal, Causalcall first standardizes the raw current measurements *X* *_raw_* using median absolute deviation (MAD). The standardization formula is defined as:

(1) $$X=\frac{c(X_{raw}-median(X_{raw}))}{median(|X_{raw}-median(X_{raw})|)}$$

where *c* is a constant.

**Figure 2:**
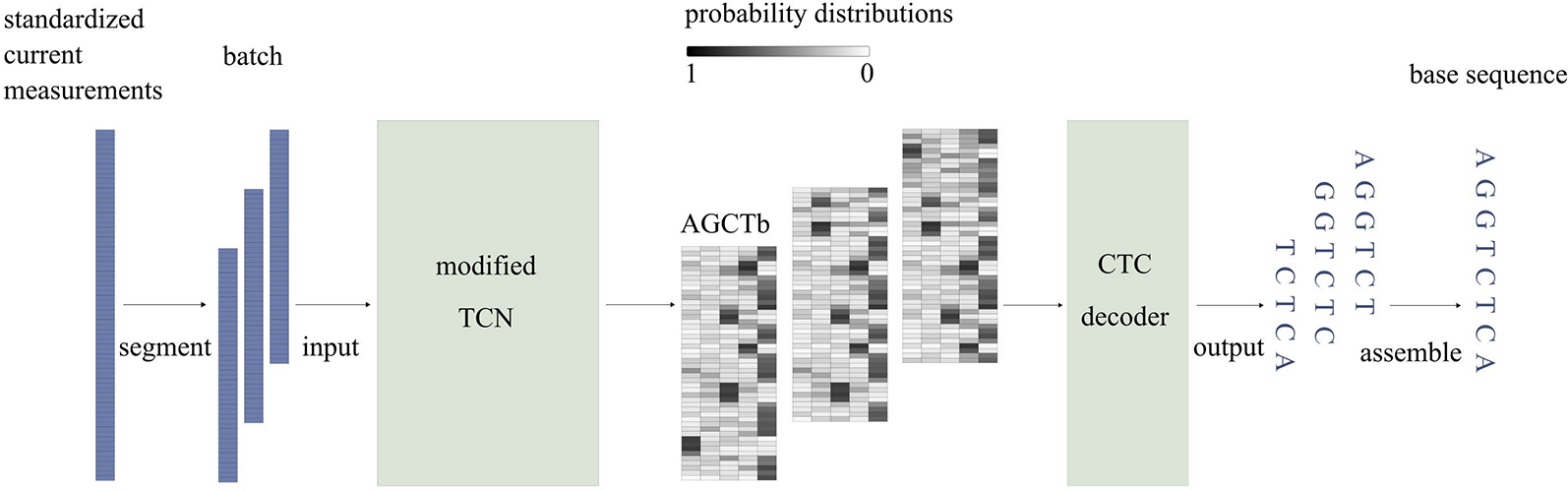
The simplified workflow of identifying the base sequence of one sequencing read. In the probability matrix, each row corresponds to a time point at the segment of current measurements. Each column corresponds to one type of base.

**Figure 2** illustrates a simplified workflow for identifying the base sequence of one sequencing read. To improve speed, current measurements are first divided into segments of length *T* (*T* = 512), using a sliding window with a step size of *d* (*d = T*/4). Then, the segments are stacked in sequence and passed to the model in batches. While passing through the model, current measurements of a segment are modeled using a modified TCN and then mapped to probability distributions, which indicate the probabilities of each base appearing at each time point. At the end of the model, the CTC decoder identifies the base sequence corresponding to the segment based on the probability distributions. Finally, Causalcall assembles base sequences of segments from the same sequencing read by overlaps and outputs the assembled sequence.

### Architecture of the TCN-Based Model

**Figure 3:**
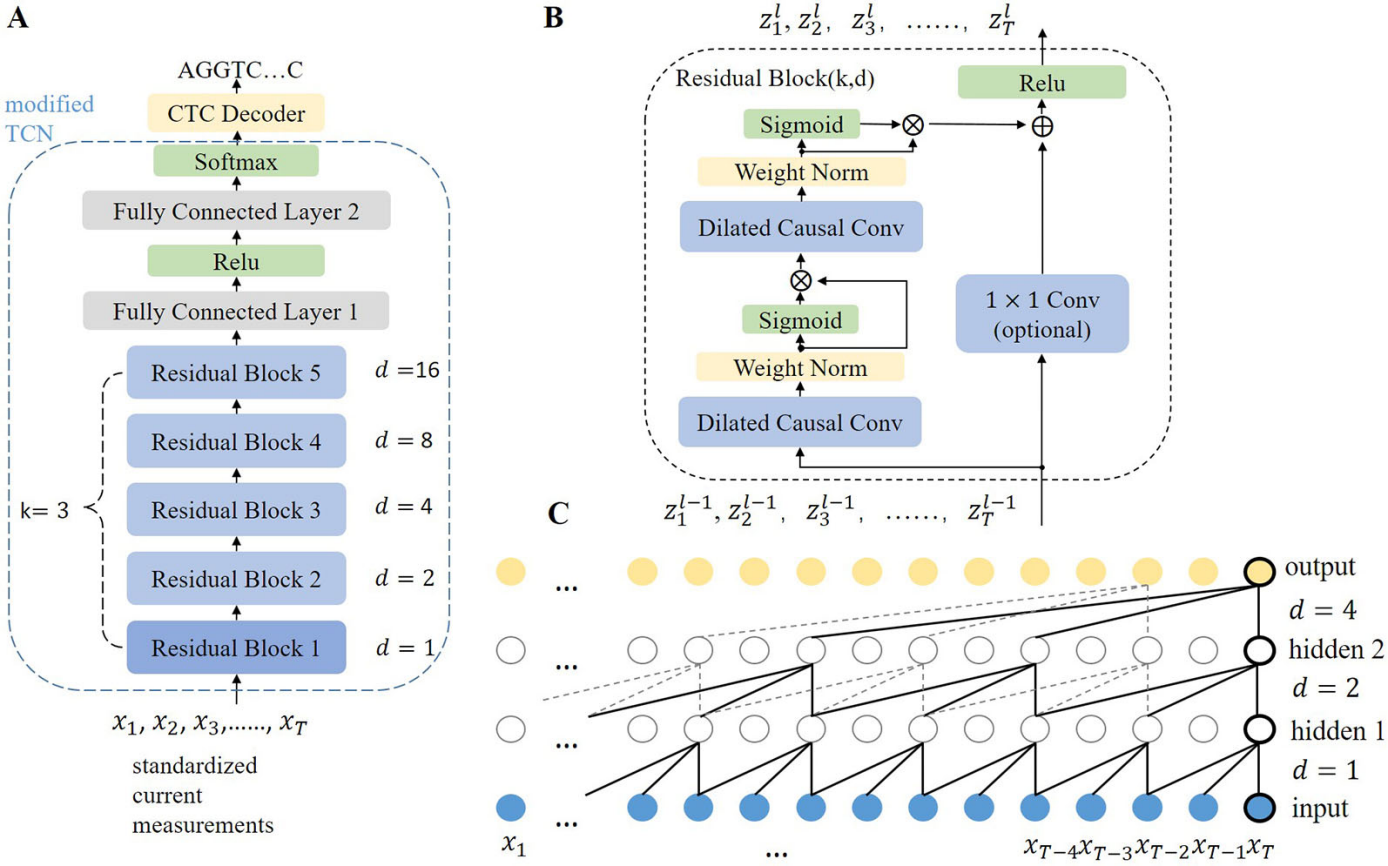
**(A)** Overall architecture of the TCN-based model. The modified TCN is formed by five stacked residual blocks with filter size *k* = 3, number of filters *n* = 256, and dilation factors *d* = 1, 2, 4, 8, 16. The output channels of the first FC layer and second FC layer are 128 and 5, respectively. The weights in the network are initialized with no bias. **(B)** Architecture of a residual block. A 1×1 convolution is implemented if the input and output of the residual block have different dimensions. *Z^l^* denotes the output of block *l*. **(C)** Dilated causal convolution network with two hidden layers. As each layer has the same length as the input layer, the convolution stride equals one. The receptive field of the convolution is computed as (*k* − 1)*d*, which means that higher layers can cover longer current segments.

As the rate of DNA movement is unstable and slower than the rate of current sampling, the base sequences may differ in length and be much shorter than the segments of current measurements. Thus, the main task of the model is to transform the segments of current measurements with fixed length *T* into base sequences with non-uniform length *M* (0 < *M* < *T*). In this study, *F* denotes the model and the transformation is written as *Y = F* (*X*), where *X*=[*x*_1_, *x*_2_,⋅⋅⋅,*x*_*T*_], *x*_*i*_∈*R* and *Y*=[*y*_1_, *y*_2_,⋅⋅⋅,*y*_*M*_], *y*_*j*_ ∈ *A*, *G*, *C*, *T*, *M* < *T*. *X* denotes a segment of current measurements with *T* time points. *Y* denotes a sequence of *M* bases. During training, *X* and *Y* are sampled from the training set *D* = {(*X* ^1^,*Y* ^1^), (*X* ^2^, *Y* ^2^),…}. Causalcall uses an end-to-end deep neural network model to learn the transformation from *X* to *Y* directly. The overall architecture and further details of the model are illustrated in **Figure 3A**. The model primarily consists of a modified TCN and a CTC decoder. The modified TCN models sequential features from the current measurements and maps them to probability distributions of bases appearing at each time point. Then, the CTC decoder implements a beam search algorithm to identify the most likely base sequence from the probability distributions.

The modified TCN is formed of five stacked residual blocks (**Figure 3B**) and two fully connected (FC) layers, which is different from the original TCN (Bai et al., 2018). The main idea in TCN architecture can be simplified to stacking dilated causal convolution layers of the same length. This scheme is illustrated in **Figure 3C**. For a given input signal sequence *X* = [*x*_1_, *x*_2_,…*x_T_*] and a filter *f*:{0,…, *k* − 1} → *R*, the dilated causal convolution operation (*C*) on the *i*th point of *X* is written as:

(2) $$C(x_{i})=\sum_{a=0}^{k-1}f(a)\cdot x_{i-a\cdot d}$$

where *d* is the dilation factor and *k* is the filter size. The output of a dilated causal convolution layer is represented by *H = C* (*X*). For the first layer, *X* denotes the input current measurements, whereas for a higher layer, it denotes the output of the former layer. Dilation factor *d* increases exponentially by 2 and the index is the number of layers.

Rather than simply stacking layers, the TCN stacks residual blocks to form a deeper architecture. We modified the original residual block (see **Figure 3B**). Each block contains two stacked dilated causal convolution layers. The two stacked layers have the same filter size (*k*), dilation factor (*d*), and number of filters (*n*). Weight normalization (Salimans and Kingma, 2016) is applied to each dilated causal convolution layer, followed by a gated linear unit (Dauphin et al., 2017) as the activation function. The formula of the gated linear unit is *H* ⊗ σ (*H*), where ⊗ means pointwise multiplication. The gated linear unit allows the model to select which features are relevant for predicting the correct bases. Then, a residual connection is applied to the activated output of the second convolution layer and the input of the block, followed by a ReLU activation function. For the fifth block, the receptive field of convolution is 32. It is sufficient to cover the current measurements that are related to the base shifted out of the pore. The stacked residual blocks act as a feature extractor to map current measurements to feature space. As the causal convolution is implemented in the time dimension, the extracted features indicate the correlation of the current measurements at different time points. Subsequently, two FC layers follow the last residual block and a softmax function is added at the end. The FC layers are used to fuse features from different channels. Then, a softmax function transforms the output of the last FC layer into a matrix of probabilities, in which each row indicates the probabilities of bases appearing at that time point.

CTC (Graves et al., 2006) is a popular approach for end-to-end sequence learning tasks. During training, it is a loss function that enables the model to directly learn the mapping from the input sequences to target sequences. It views the output of the modified TCN as the probability distributions over all possible base sequences conditioned on the input segment of the current measurements. Then, a loss function is computed based on the probability distributions. Given input segment *X* = [*x*_1_, *x*_2_,…*x_T_*] and label sequence *Y* = [*y*_1_, *y*_2_,…, y_M_], *W* denotes the transformation of the modified TCN. The output of the modified TCN is written as [*O*_1_, *O*_2_,…, *O_T_*] = *W* ([*x*_1_, *x*_2_, …, *x_T_*]), where *O_t_* is a vector of length 5 that indicates the probability of each symbol (*A*, *G*, *C*, *T*, *blank*) appearing at time point *t*. *Blank* denotes a symbol introduced by CTC. It acts as a separator to separate adjacent bases in label sequence and will not appear in the output sequence. The probability of label *Y* conditioned on *X* is defined as:

(3) $$P(Y|X)=\sum_{s\in B^{-1}(Y)}P(s|X),\;P(s|X)=\overset{T}{\underset{t=1}{\Pi }}O_{t}^{s_{t}},\;\forall s\in Y^{\prime }$$

where *s*=[*s*_1_, *s*_2_, ⋅⋅⋅,*s*_*T*_], *s*_*t*_∈{*A*, *G*, *C*, *T*, *blank*} denotes the sequence of possible symbols conditioned on *X*. $O_{t}^{s_{t}}$ is a value output by the modified TCN at time point *t*, which indicates the probability of symbol *s_t_* appearing at time point *t*. *Yꞌ* is a set of all possible symbol sequences (*s*) with length *T*. Additionally, *B* denotes a many-to-one mapping function that first merges the consecutively repeated symbols and then removes all blanks in *s*. Thus, the possible symbol sequences can be mapped to a shorter base sequence. The model is trained with the goal of optimizing loss function *L_ctc_* = − log*P*(*Y*|*X*). The Adam optimizer with an initial learning rate of 0.004 is used to optimize the loss.

During testing, based on the premise of CTC, a beam search or greedy search algorithm is implemented to identify the most likely base sequence directly. In the greedy search algorithm, symbols with the highest probability are selected at each time point to form a symbol sequence. Then, the symbol sequence is transformed into a base sequence according to the many-to-one mapping [e.g., *Y = B* (*s*) = *B*([*A*, *A*,−, −,*G*, −,*C*]) = [*A*, *G*, *C*], − *denotes blank*]. The beam search algorithm is based on a similar idea, but it maintains multiple sequences during decoding. The sequence with the highest score will be the final base sequence.

### Training

We train Causalcall on the mixed training set. To prevent overfitting, the model is evaluated every 10 iterations of training on a separate validation set. Before training, the current measurements are standardized using MAD and are divided into segments with a fixed length (as described in the *Data Preparation* section). The segments are fed into the model in batches. We use a segment length of 512 and a batch size of 256. During training, the training set and validation set are shuffled after each epoch. The learning rate is stepped down as the number of iterations increases. The entire model is trained on an NVIDIA 1080ti GPU with 12 GB memory, and it takes nearly 3 days to converge.

### Metrics for Basecalling Evaluation

To evaluate the performance of basecallers, we mapped basecalling results to the reference genome. First, the base sequences from different basecallers are mapped to the reference genome using minimap2 (Li, 2018) with the parameter *map-ont*. Mapping results are stored in SAM files and analyzed by a japsa^1^ error analysis tool (*jsa.hts.errorAnalysis*) to assess the identity rate and error rates, including insertions, deletions, and mismatches, of each mapping. Identity indicates correct alignments between the base sequence and the reference genome. The identity rate is computed as $\frac{number\;of\;matched\;bases}{number\;of\;bases\;in\;reference}$. It is a metric for the accuracy of basecalling. The insertion rate, deletion rate, and mismatch rate are computed as the numbers of inserted bases, deleted bases, and mismatched bases divided by the number of bases in the reference genome, respectively. Mismatch indicates incorrect alignments. Deletion/insertion indicates that a base deletion/insertion occurs in the base sequence relative to the reference genome. They are metrics for basecalling errors. We also computed the total error rate by summing up rates of insertions, deletions, and mismatches. In addition, the speed of basecalling is also an important metric to evaluate the performance of basecallers. The speed is calculated by the number of outputted bases divided by the running time. Higher values of identity rate and speed reflect better performance, while for the other metrics, lower values are better.

### Genome Assembly and Error Analysis

We also used the quality of the assembled genome to evaluate reads from different basecallers. In this study, base sequences of *K. pneumoniae* are assembled using the assembly pipeline introduced by Rebaler (Wick et al., 2019). The samples of *K. pneumoniae* contain no plasmids and the sequencing depth is over 100× on average, making them easier to assemble. The assembly pipeline contains two main steps. First, based on the mapping results of minimap2, the matched parts in the reference genome are replaced with read sequences to generate a draft assembled genome. Second, the draft assembled genome is polished with 10 rounds of Racon (Vaser et al., 2017). The pipeline ensures that the assembled genome and the reference genome are similar in size. For efficient mapping, the assembled genome is first segmented into 10 kbp pieces. Then, these pieces are mapped to the reference genome using minimap2 with the parameter *ax asm*. The rates of identity, deletion, insertion, and mismatch are determined from the mapping results using japsa. The homopolymer and methylation of DNA are two common factors that may cause base deletion and base substitution (Wei et al., 2017. Taking into account their effects, mapping errors of the assembled genomes are further classified into six types in the context of the reference genome. Based on the assembled genomes of *K. pneumoniae*, the error analysis method described in Rebaler is implemented. Pieces of the assembled genome are mapped to the reference genome using the *nucmer* command of MUMmer v4.0.0 (Kurtz et al., 2004). Then, the *delta-filter* command and the *show-snps* command are used to detect single-nucleotide polymorphism (SNP). Based on the results of mapping and SNP detection, errors are classified into homopolymer deletion, homopolymer insertion, Dcm error, insertion, deletion, and substitution. Homopolymer insertion and homopolymer deletion represent insertion and deletion errors, respectively, that occur in regions of homopolymers with more than two bases. Dcm error represents errors that occur in Dcm-methylation motifs (CCTGG or CCAGG). Errors not in the former three categories are classified as ordinary deletion, insertion, or substitution.

## Results

### Benchmark Methods and Experimental Details

In this study, we evaluate the performance of Causalcall, Chiron v0.4.2, Flappie v1.1.0, and Guppy v3.1.5. As Causalcall and Chiron are both developed on TensorFlow using Python and run on a GPU, we focus on comparing them. For an effective comparison, Chiron is retrained with default parameters on the mixed training set and it takes 2.5 days to converge. The retrained model is called DNAre, and the default model of Chiron is called DNAde. Flappie and Guppy are state-of-the-art official basecallers. The two official basecallers are trained with non-public datasets that are different from ours. Alternatively, the official basecallers are used as references in performance comparison. To demonstrate the effect of training data, Chiron is run with a default model and a retrained model, respectively. Flappie is run with 20 CPU threads. Guppy, Chiron, and Causalcall are run on an NVIDIA 1080ti GPU with 12 GB memory. As for the basecalling parameters, Causalcall uses a segment length of 512, batch size of 256, and sliding window stride of 118. The other basecallers are run with the default parameters.

### Evaluation Results of Basecallers

The results of evaluating the four basecallers on the four testing sets are summarized in **Table 1**. Compared with Chiron, Causalcall achieves a higher identity rate and a lower error rate on the four species. In terms of three mapping errors, Causalcall has lower insertion and mismatch rates. In particular, Causalcall has the lowest insertion rate on phage and human. These results show that Causalcall effectively learned the base-related patterns hidden in the current measurements. Furthermore, they indicate that the TCN-based model of Causalcall has greater potential for improving the accuracy of basecalling than the RNN-based model of Chiron. Regarding the speed of basecalling, Causalcall reaches 7,000 bases per second on average, which is nearly three times faster than Chiron. This improvement in speed indicates that the TCN-based model indeed speeds up basecalling, resulting from the matrix computation of dilated causal convolution. In addition, Causalcall adopts longer segments of current measurements with lower overlap ratio compared with Chiron. This also contributes to the improvement in speed. As we mentioned in the previous section, the training data of Guppy and Flappie are not publicly available. It is thus difficult to say whether the training data or their RNN-based models contribute to the good performance. However, we observed that the training data indeed have a significant impact on the performance of basecallers. As shown in **Table 1**, Chiron (DNAre) achieves a 1.78% higher identity rate and a 1.85% lower error rate than Chiron (DNAde) on average, which corroborates the effect of the training data. Flappie is programmed with C language and accelerated by multiple threads of a CPU. The speed of Flappie depends on the number of threads and the state of the CPU. As such, the speed could fluctuate greatly. Experimental results show that Causalcall is as fast as Flappie that with 20 CPU threads. Guppy is a developed software in binary version. We do not know its inner software engineering technologies used for acceleration, as it is not open source. Guppy works much faster than Causalcall at present, but Causalcall can be further accelerated by reprogramming with C language and integrating with acceleration technologies such as parallel computing. The results above demonstrate that the TCN-based model of Causalcall could improve the accuracy and speed of basecalling.

**Table 1:** Performance of Causalcall, Chiron v0.4.2 (DNAde), Chiron v0.4.2 (DNAre), Guppy v3.1.5, and Flappie v1.1.0 on the four testing sets.

Species	Basecaller	Deletion (%)	Insertion (%)	Mismatch (%)	Identity (%)	Error (%)	Speed (bps)
Lambda phage	Causalcall	6.48	1.84	4.30	89.21	12.62	7385
	Chiron (DNAde)	8.20	2.27	5.77	86.03	16.24	2568
	Chiron (DNAre)	6.86	2.22	4.71	88.43	13.79	2721
	Guppy	4.60	2.02	3.00	92.40	9.62	379883
	Flappie	5.01	2.28	3.50	91.50	10.79	9398
*E. coli*	Causalcall	5.95	2.07	4.57	89.48	12.59	7172
	Chiron (DNAde)	7.07	2.47	6.04	86.89	15.58	2485
	Chiron (DNAre)	5.91	2.34	4.65	89.44	12.90	2761
	Guppy	4.06	1.97	3.02	92.92	9.05	363389
	Flappie	4.60	2.28	3.60	91.79	10.48	6678
Human	Causalcall	8.06	2.27	5.06	86.88	15.39	6548
	Chiron (DNAde)	8.49	2.92	5.4^1^	86.10	16.82	2045
	Chiron (DNAre)	7.76	2.98	5.56	86.68	16.30	1992
	Guppy	4.78	2.46	2.86	92.35	10.10	349665
	Flappie	5.33	2.67	3.35	91.32	11.35	7212
*K. pneumoniae*	Causalcall	5.58	4.82	6.29	88.12	16.69	6657
	Chiron (DNAde)	5.70	6.41	7.92	86.38	20.03	2540
	Chiron (DNAre)	5.13	6.26	6.9	87.98	18.29	2446
	Guppy	4.10	4.16	4.49	91.41	12.75	305153
	Flappie	5.06	4.26	5.42	89.52	14.74	6400

1 Chiron (DNAde) and Chiron (DNAre) represent the default and the retrained Chiron, respectively.

Identify rate of the mapping is the metric for basecalling accuracy [the higher the better, identify rate = 1 – (deletion rate + mismatch rate)]. Error rate is calculated by summing the rates of deletion, insertion, and mismatch (the lower the better).

**Figure 4:**
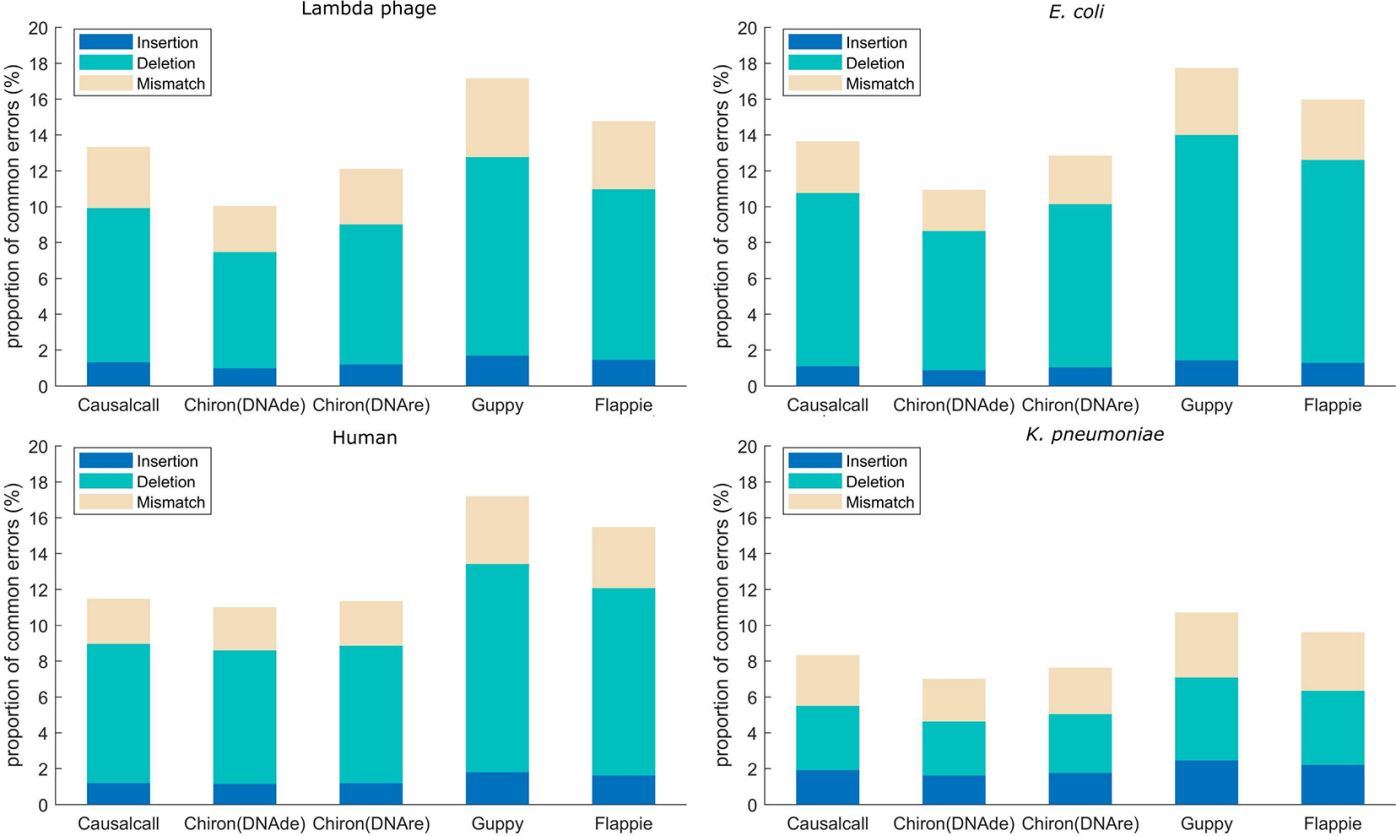
Proportion of common mapping errors relative to total mapping errors for base sequences from each basecaller.

Mapping errors of base sequences are related not only to errors caused by basecalling methods but also to errors caused by the nanopore device and modification or mutation of DNA. In this paper, we assume that the mapping errors in common among different basecallers are more likely to be caused by the nanopore device or DNA samples, whereas others are more likely to be caused by the basecalling methods. Accordingly, we analyzed the overlaps of mapping errors of different basecallers. For each basecaller, we computed the proportion of common mapping errors relative to total mapping errors. The mapping errors are divided into three types: insertion, deletion, and mismatch. For example, as for one sequencing read in the testing set, if the resulting base sequences from the four basecallers have base insertions at the same site on the reference genome, this site is determined to have a common insertion error. Similarly, common deletion and common mismatch errors are counted in this manner. For each basecaller, the proportion of common errors (insertion, deletion, or mismatch) is computed as the number of common errors divided by the number of total errors of that basecaller. The results of the error overlap analysis are shown in **Figure 4**. A higher proportion of common errors indicates the better performance of the basecaller. Causalcall achieves higher common error proportions than Chiron on all species. This indicates the better performance of Causalcall than Chiron as there are fewer errors introduced by itself during basecalling. Further, it demonstrates that the TCN-based model could properly model current measurements under the sequence-to-sequence learning strategy. Similar to the previous paragraph, the results of Flappie and Guppy act as references. Additionally, we observed that the common error proportions of lambda phage, *E. coli*, and human are higher than that of *K. pneumoniae*. The main reason for this is that Causalcall and Chiron have learned feature distributions of current measurements about these three species. This indicates that the multi-species training data could help to improve the accuracy of basecalling.

### Effects of Basecallers on the Quality of Genome Assembly

Sequences of long nanopore reads have been shown to play a significant role in high-quality genome assembly. We analyze the effects of base sequences from different basecallers on the qualities of mapping of the assembled genome to the reference genome. We assemble the base sequences of *K. pneumoniae* by first generating a draft assembled genome based on the alignments of the base sequences to the reference genome. Then, we polish the draft assembled genome with 10 rounds of Racon (see the *Materials and Methods* section). To assess the quality of the assembled genome, we use minimap2 to map pieces of the assembled genome to the reference genome. Based on the mapping results, we count the rates of identity, deletion, insertion, and mismatch, which act as assessment metrics.

Mapping qualities of the assembled genomes corresponding to different basecallers are shown in **Table 2**. The assembled genome of Causalcall shows high quality with an identity rate of 99.81%, which is much higher than those of Chiron (DNAde) and Flappie. The assembled genome of Causalcall also has a 0.04% lower error rate than that of Chiron (DNAre), although it has a 0.04% lower identity rate. In addition, the assembled genome of Causalcall has the lowest insertion and mismatch rates among the five assembled genomes. The main reason for this is that most of the insertion and mismatch errors in base sequences of Causalcall have been corrected by Racon in the process of polishing. These results further indicate the high utility of Causalcall in reference-based genome assembly.

**Table 2:** Mapping qualities of the assembled genomes of *K. pneumoniae* corresponding to different basecallers. Error rate is calculated as that in **Table 1**.

Basecaller	Deletion (%)	Insertion (%)	Mismatch (%)	Identity (%)	Error (%)
Causalcall	0.17	0.01	0.02	99.81	0.20
Chiron (DNAde)	0.09	0.28	0.27	99.64	0.64
Chiron (DNAre)	0.10	0.09	0.05	99.85	0.24
Guppy	0.09	0.01	0.03	99.88	0.13
Flappie	0.21	0.01	0.10	99.69	0.32

Considering the effects of homopolymers and Dcm-methylation, we further analyzed six assembly errors of the assembled genomes. For precise mapping at the genome level and SNP detection at the base level, we used the commands of MUMmer v4.0.0 (see the *Materials and Methods* section). Based on the results of mapping and SNP detection, errors are classified into homopolymer deletion, homopolymer insertion, Dcm error, insertion, deletion, and substitution. The six error rates are additional metrics to evaluate basecallers.

**Figure 5:**
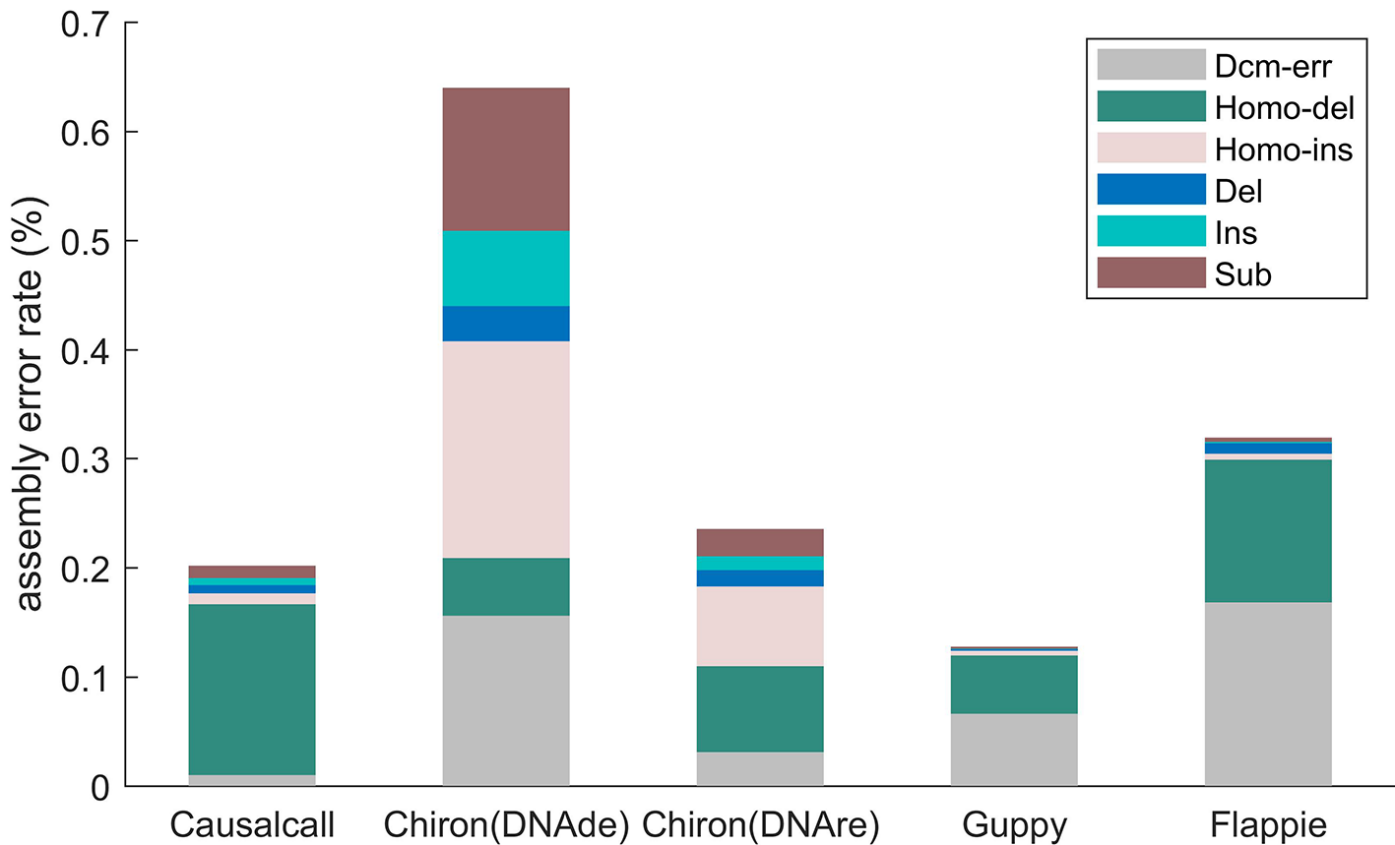
Assembly error rates of different basecallers on *K. pneumoniae*, classified by type. Errors of mapping the assembled genome to the reference genome are classified into six types. Homopolymer insertion/deletion (Homo-ins/Homo-del) represents errors that occur in homopolymers with more than two bases. Dcm error (Dcm-err) represents errors that occur in Dcm-methylation motifs (CCTGG/CCAGG). Deletion (Del), insertion (Ins), and substitution (Sub) are ordinary error types. Each type of error rate is computed as the number of errors in the category divided by the length of the reference sequence.

**Figure 5** illustrates the assembly error rates of base sequences from different basecallers. The assembled genome of Causalcall achieves the second lowest assembly error rate in total, among the five assembled genomes. Compared with Chiron, the assembled genome of Causalcall has lower rates on all assembly errors, except for homopolymer deletion. Homopolymer deletion is the most common assembly error of Causalcall and Flappie. The main reason for this is that it is difficult for Racon to correct errors in long homopolymers. It is worth noting that the assembled genome of Causalcall has the lowest error rate in Dcm-methylation, which is the main type of assembly error of Guppy and Flappie. This superior result indicates that Causalcall has exploited representative features of DNA methylation from the training data, resulting in a better ability to correctly identify bases from the current measurements of methylated DNA.

## Discussion

We proposed Causalcall for nanopore basecalling. It uses a TCN-based model to directly identify base sequences from current measurements in high speed. The core component of TCN is the dilated causal convolution introduced by WaveNet. As described in a recent publication, Wavenano uses bidirectional WaveNets for basecalling in the manner of classification. It relies on the precise labeling of each current measurement in training data. However, it is difficult to precisely label each current measurement, resulting from the noise in the current signal and uneven flow of DNA. Causalcall is designed in a manner of sequence labeling. Therefore, it does not rely on precise label of each current measurement in training. The TCN is tailored to combine with a CTC decoder to properly deal with long-range temporal dependencies of current measurements and non-uniform lengths of base sequences. As a result, the proposed model can directly identify base sequences of varying lengths from the segments of current measurements in long time series. The CTC decoder helps to realize sequence-to-sequence learning. A gated linear unit is added to help the model select important features. These designs mitigate the impact of imprecise alignments between current measurements to reference bases during labeling of the training data. Moreover, they promote the performance of the network in modeling current measurements.

Trained on a mixed training set, Causalcall shows better performance on samples of multiple species than Chiron. Among the three types of mapping errors, base deletions are the most common errors for the four basecallers. Such deletions are mainly caused by long-term and non-fluctuating current signals of homopolymers. To properly deal with this problem, Guppy and Flappie take a flip-flop algorithm to distinguish the repeated bases of homopolymers. Following this idea, we could modify the label sequence of training data by marking consecutively repeated bases with two states and retraining the TCN-based model. This could be a way of decreasing the deletion rate. We will verify this idea in future work.

## Conclusion

We proposed Causalcall, which uses an end-to-end TCN-based model for accurate and fast nanopore basecalling. The TCN-based model is well designed based on a modified TCN and a CTC decoder. Thus, it could efficiently identify base sequences of different lengths from the current measurements in long time series. Different from the ordinary RNN-based models, the TCN-based model could speed up basecalling by matrix computation. The highly parallelizable architecture allows the model to be further accelerated by technologies such as parallel computing. Trained with a mixed training set, Causalcall shows good performance on samples of trained as well as untrained species. Compared with the RNN-based Chiron, Causalcall achieves higher identity rates, lower error rates, and nearly three times higher speed on the four testing sets. Such improvements in performance demonstrate that the TCN-based model has great potential in improving basecalling accuracy and speed. Additionally, the assembled genome of Causalcall shows high quality with the lowest error rate in Dcm-methylation motifs. It demonstrates the high utility of Causalcall in reference-based genome assembly. It also indicates the ability of the TCN-based model to identify bases from the current measurements of methylated DNA.

## Data Availability Statement

The sequencing data and reference genomes of lambda phage and *E. coli* (Teng et al., 2018) can be downloaded from https://data.genomicsresearch.org/Projects/train_set_all. The sequencing data and reference genome of *K. pneumoniae* INF042 (Wick et al., 2019) can be found in figshare with the identifiers https://doi.org/10.26180/5c5a5fa08bbee and https://doi.org/10.26180/5c5a5fcf72e40. The human sequencing data (Jain et al., 2018) can be downloaded from https://github.com/nanopore-wgs-consortium/NA12878/blob/master/nanopore-human-genome/rel_3_4.md. GRCh38 with decoys can be downloaded from ftp://ftp.1000genomes.ebi.ac.uk/vol1/ftp/technical/reference/GRCh38_reference_genome/. The source code of Causalcall is available via https://github.com/scutbioinformatic/causalcall.

## Author Contributions

HC conceived, designed, and supervised all parts of the project. JZ designed the method, performed experiments, and wrote the manuscript. HP and HW contributed to discussions and editing of the paper. TA proposed guiding suggestions for the editing of the paper. YZ contributed a lot in conducting the experiments and paper revision. All authors read and approved the final version of the manuscript.

## Funding

This work was partially supported by the National Natural Science Foundation of China (61771007), Applied Science and Technology Research and Development Project of Guangdong Province (2016B010127003), and Health Medical Collaborative Innovation Project of Guangzhou City (201803010021).

## Conflict of Interest

The authors declare that the research was carried out in the absence of any commercial or financial relationships that could be construed as a potential conflict of interest.

## Footnotes

1 japsa toolkit:https://github.com/mdcao/japsa.
